# Potential Human Health Benefits of *Phaseolus vulgaris* L. var Venanzio: Effects on Cancer Cell Growth and Inflammation

**DOI:** 10.3390/nu16152534

**Published:** 2024-08-02

**Authors:** Clizia Bernardi, Giorgio Cappellucci, Giulia Baini, Anna Maria Aloisi, Federica Finetti, Lorenza Trabalzini

**Affiliations:** 1 Department of Biotechnology, Chemistry and Pharmacy, University of Siena, Via Aldo Moro 2, 53100 Siena, Italy; clizia.bernardi@student.unisi.it (C.B.); lorenza.trabalzini@unisi.it (L.T.); 2 Department of Physical Sciences, Earth and Environment, University of Siena, Via Laterina 8, 53100 Siena, Italy; giorgi.cappellucci@unisi.it (G.C.); giulia.baini2@unisi.it (G.B.); 3 Department of Medicine, Surgery and Neuroscience, University of Siena, Via Aldo Moro 2, 53100 Siena, Italy; annamaria.aloisi@unisi.it

**Keywords:** common beans, *Phaseolus vulgaris*, colon cancer, inflammation, prostaglandin E2, interleukin 1β, NOX1, biodiversity, in vitro gastrointestinal digestion, waste recovery

## Abstract

It is widely recognized that foods, biodiversity, and human health are strongly interconnected, and many efforts have been made to understand the nutraceutical value of diet. In particular, diet can affect the progression of intestinal diseases, including inflammatory bowel disease (IBD) and intestinal cancer. In this context, we studied the anti-inflammatory and antioxidant activities of extracts obtained from a local endangered variety of *Phaseolus vulgaris* L. (Fagiola di Venanzio, FV). Using in vitro intestinal cell models, we evaluated the activity of three different extracts: soaking water, cooking water, and the bioaccessible fraction obtained after mimicking the traditional cooking procedure and gastrointestinal digestion. We demonstrated that FV extracts reduce inflammation and oxidative stress prompted by interleukin 1β through the inhibition of cyclooxygenase 2 expression and prostaglandin E2 production and through the reduction in reactive oxygen species production and NOX1 levels. The reported data outline the importance of diet in the prevention of human inflammatory diseases. Moreover, they strongly support the necessity to safeguard local biodiversity as a source of bioactive compounds.

## 1. Introduction

The importance of bioactive compounds in foods and their beneficial impacts in the prevention and treatment of several human diseases is widely recognized. In addition, One Health approaches outline the interdependence among the health of humans, animals, and ecosystems [1]. As suggested by the World Health Organization, nutrition, biodiversity, and human health are strongly interconnected. The nutritional values of foods and the varieties/cultivars/breeds of the same food can be very different, modifying micronutrient availability in the diet and influencing the health of the local population. Maintaining high levels of biodiversity is a fundamental requirement to ensure adequate average nutrient intake levels in the diet. In this scenario, this paper describes the potential human health benefit of *Phaseolus vulgaris* L. var Venanzio, FV, a local endangered bean variety grown in a restricted area of the municipality of Murlo, Siena, Tuscany, recognized by Regione Toscana as a specific variety in 2017 (N. VE_145 20 December 2017) [2].

It is commonly known that the consumption of beans promotes health benefits in connection with obesity [3,4], diabetes mellitus [5], cardiovascular disease [6,7], and cancer [2,8,9,10,11,12,13]. Beans regulate gut health by inducing beneficial changes in the gut microbiota profile and activity [14,15]. Moreover, the phenolic compounds present in beans induce gut health by enhancing the integrity of the gut barrier [16,17], modulating the microbiota community [18,19,20,21], attenuating immune responses [22,23,24], and reducing oxidative stress [25,26]. Collectively, the diverse gut-health-associated targets of bioactive compounds present in beans may ameliorate the clinical history of chronic diseases including those associated with inflammatory processes.

Colorectal cancer (CRC) is a widespread disease engendered and fueled by inflammation [27]. Many studies indicate that an elevated inflammatory response in inflammatory bowel disease (IBD) is an independent factor for the induction of colitis-associated colon cancer [28]. IBD increases the possibility to develop CRC, and high levels of inflammation are associated with a similar increase in the occurrence of CRC [29]. This suggests that controlling inflammation may be an effective strategy to prevent the development and progression of CRC.

Plant-based eating patterns support health and disease relief through nutritional composition and bioactive compounds. A strong relationship between dietary habits and the risk of intestinal diseases such as IBD and CRC is now consolidated. There are some indications of the beneficial role of the consumption of beans in preventing the development of CRC, [9,30,31]. Several studies on in vitro and in vivo models support these observations [2,8,10,11,12,32,33,34]. It has been reported that different types of *P. vulgaris* bean extracts have antiproliferative and anti-inflammatory activity and induce apoptosis and autophagy in cancer cells [8,35,36,37,38].

We previously reported that the aqueous extract of FV contains high levels of proteins, sugars, and polyphenols, and exerts antioxidant, anti-inflammatory, and antiproliferative activities in colon cancer cell models [2]. In the present study, we further investigated the anti-inflammatory potential of FV in colon cancer cell models by mimicking the traditional bean cooking procedure and carrying out an in vitro gastrointestinal digestion of cooked beans. Bean soaking water (SW), cooking water (CW), and the post-digestion bioaccessible fraction (BF) were analyzed to establish their activity in the inhibition of molecular pathways linked to colon cancer cell growth prompted by inflammatory mediators and oxidative stress.

The results of this work provide preliminary evidence that including beans in the diet may affect the inflammatory molecular pathways involved in the development of colon cancer, thus supporting the importance of bean consumption for human health.

Furthermore, we propose that bean soaking water, which is generally discarded and represents a considerable waste product in bean industrial production processes, may constitute a source of bioactive compounds to be used for the preparation of food supplements as well as in pharmaceutical and nutraceutical preparations.

## 2. Materials and Methods

### 2.1. Preparation of P. vulgaris var. Venanzio (FV) Extracts

*FV beans* were first subjected to soaking and then to cooking and in vitro digestion. Briefly, 5 g of beans were soaked for several hours (overnight) in water (100 mL). The resulting water (soaking water, SW) was saved for further analysis. Then, 5 g of soaked beans were cooked for 3 h in water (100 mL). The resulting water (cooking water, CW) was collected. The digestion phase (oral, gastric, and intestinal) was performed following INFOGEST protocol 2.0 [39], slightly modified as described earlier [40]. The fraction reproducing the one able to be absorbed by the epithelium (bioaccessible fraction, BF) was collected and stored at −80 °C; the blank solution is described in [8].

### 2.2. Analysis of Polyphenolic

Chemical characterization of the polyphenolic profile of the CW and BF fractions was performed by HPLC-DAD analysis using a Shimadzu Prominence LC 2030 3D instrument equipped with a Bondapak® C18 column, 10 µm, 125 Å, 3.9 mm × 300 mm column (Waters Corporation, Milford, MA, USA) as previously described in detail in [2].

### 2.3. Cell Culture 

HT29 colorectal adenocarcinoma cells (ATCC, Rockville, MD, USA) and HCT116 colorectal carcinoma cells (ATCC, Rockville, MD, USA) were cultured as previously described in detail in [2,41].

### 2.4. MTT Assay 

Then, 3.5 × 10^3^ HT29 or 2.5 × 10^3^ HCT116 were tested as previously described in detail in [42]. Cells were treated with SW, CW, or BF (1, 10, and 100 µg/mL), or with GKT137831 (5 µM) (Cayman Chemical, Ann Arbor, MI, USA) in the presence or the absence of IL1β (10 ng/mL) (ReliaTech GmbH, Wolfenbüttel, Germany) and PGE2 (1 µM) (Sigma-Aldrich, St. Louis, MI, USA).

### 2.5. Western Blotting Analysis 

Next, 3.5 × 10^5^ cells/well were grown for 24 h in a 60 mm dish in medium with 10% serum, starved for 24 h in medium with 0.1% serum, and finally treated with FV (1, 10, 100 µg/mL) or the blank solution, with or without IL1β (10 ng/mL) and PGE2 (1 µM) or with GKT137831 (5 µM) with or without IL1β (10 ng/mL). After 48 h, the protein extracts were prepared by lysing cells in a precooled radioimmunoprecipitation assay (RIPA) buffer (Cell Signaling Technology, Danvers, MA, USA). The protein concentration of the supernatant of cell lysates obtained after centrifugation at 13,000× *g* for 15 min at 4 °C was determined by using the BCA method (BCA protein assay kit, Euroclone, Pero, Italy). Equal amounts of proteins (50 µg) were analyzed by sodium dodecyl sulfate (SDS)-polyacrylamide gel electrophoresis (PAGE) and Western blotting analysis, as previously described [43]. The following primary antibodies were used: anti-LC3, anti-NOX1, anti-COX2, anti-Caspase3, anti-GAPDH, and anti-β-actin (Cell Signaling Technology). Immunoreactive proteins were visualized by using an enhanced chemiluminescence (ECL) detection system (Euroclone). Images were digitalized with Image Quant LAS4000 (GE Healthcare Europe GmbH, Milano, Italy). Immunoreactive bands were quantified by densitometry using the ImageJ software 1.52a Java 1.8.0_112 (64-bit) (an open-source image processing program, National Institutes of Health, Bethesda, MD, USA).

### 2.6. Clonogenic Assay 

Then, 3.5 × 10^2^ HT29 cells were seeded in 24 multi-well plates in the presence of a medium containing 10% serum. After adhesion, cells were exposed to SW, CW, and BF (1, 10, 100 µg/mL) in 1% serum with or without IL1β (10 ng/mL) and PGE2 (1 µM). After 10 days, the colonies were fixed and counted [42].

### 2.7. Trypan Blue Assay 

Following this, 7.5 × 10^5^ HT29 cells were suspended in 1 ml of RPMI containing 0.1% serum and exposed to 100 μg/mL BF or blank solution. After 48 h, 0.4% trypan blue was added to cell suspension (1/1) h, and cell counting was performed with the LUNA-II Automated Cell Counter (Logos Biosystems, Anyang, Republic of Korea).

### 2.8. Immunofluorescence Assay 

Next, 7.5 × 10^4^ HT29 cells/well cells were plated on glass coverslips into 24 multi-well plates in RPMI containing 10% serum and incubated for 24 h. Cells were then exposed to 100 µg/mL BF for 24 h and fixed in cold acetone (5 min). Staining with anti-LC3B antibody was performed overnight at 4 °C, after 1 h of incubation with 3% bovine serum albumin (BSA). Cells were detected after 1 h of incubation with an anti-rabbit secondary antibody (1:150, Alexa Fluor 488, ThermoFisher, Waltham, MA, USA) and a further 5 min with 0.1 µg/mL DAPI (Cell Signaling Technology). Immunofluorescent staining was visualized with the ECLIPSE Ts2 microscope and images were captured with NIS-Elements software D 5.30.02 64-bit (Nikon, Minato City, Tokyo, Japan) [41].

### 2.9. Annexin V-FITC Staining 

Annexin V-FITC staining was performed as previously reported [8].

### 2.10. Senescence Assay 

For the senescence assay, 7.5 × 10^4^ HT29 cells/well were plated in 24 multi-well plates in RMPI medium containing 10% of serum. After adhesion, cells were exposed for 24 h to 1, 10, and 100 µg/mL of FV extracts. The senescence β-galactosidase staining kit (Cell Signaling Technology) was used to determine the senescent cells.

### 2.11. Determination of Reactive Oxygen Species (ROS) 

To determine ROS, 5.0 × 10^4^ cells/well (HT29) were plated in 24 well multi plate and exposed to 10 ng/mL of IL1β or 1 µM PGE2, where indicated cells were pre-treated for 1 h with FV extracts (10 µg/mL) or GKT137831 (5 µM). After 24 h, cells were trypsinized and suspended with 10 µM of 2,-7-dichlorodihydrofluorescein diacetate (DCFH_2_-DA, ThermoFisher) for 15 min. Cells were spun and resuspended with PBS. ROS levels were measured photometrically with a CLARIOstar microplate reader (BMG LABTECH) (excitation/emission 495/527).

### 2.12. Determination of PGE2 by ELISA 

HT29 (5.0 × 10^4^ cells/well) were plated in 24 multi-well plates. After starvation, cells were exposed to 10 μg/mL of FV extracts or GKT137831 (5 µM) for 1 h. Cells were then treated with 10 ng/mL of IL1β for 48 h. The PGE2 concentration in the supernatants was determined by using the Prostaglandin E2 Express ELISA Kit (Cayman Chemical, Ann Arbor, MI, USA) [44].

### 2.13. RT-PCR

Then, 3.5 × 10^5^ cells/well (HT29) were plated in a 60 mm dish in medium containing 10% serum. After starvation, cells were treated with 10 µg/mL of FV extracts for 1 h and then exposed to IL1β (10 ng/mL) or PGE2 (1 µM) for 24 h. Cells were then lysed with an RNA lysis buffer (Zymo Research, Sunnyvale, CA, USA) and the RNA purification was performed with the Quick-RNA Miniprep Kit (Zymo Research). The iScript cDNA Synthesis kit (BioRad, Hong Kong, China) was used for reverse transcription. In total, 50 ng/μL of cDNA samples were used to amplify COX2 and NOX1 with gene-specific primers (Bio-Fab Research, Roma, Italy). All real-time PCR reactions were performed using the Rotor-Gene Q (Qiagen, Hong Kong, China) and the amplifications were performed using the Luna Universal qPCR Master Mix (New England BioLabs, Ipswich, MA, USA) applying the following conditions: an initial denaturation step at 95 °C for 1 min, 45 cycles at 95 °C for 15 s, 60 °C for 30 s, and 95 °C for 10 s. The relative quantification of gene expression was determined using the 2^−ΔΔCt^ method [45].

### 2.14. Statistical Analysis 

Data are expressed as mean ± standard deviation (SD). Statistical analysis was performed using Student’s *t*-test, one-way ANOVA, or Tukey’s multiple comparisons test (GraphPad Prism 8.4.3). Any differences in the dataset of *p* < 0.05 were considered statistically significant.

## 3. Results

### 3.1. Activity of P. vulgaris Extracts on Colon Cancer Cells

As previously reported for other varieties of *P. vulgaris* [8], in this study we used different fractions obtained after soaking, cooking, and in vitro digestion of FV beans. The chemical characterization of the fractions is currently underway. However, preliminary data on polyphenol content [8] indicate that the pre-digestion fraction (CW) contains polyphenols that are lost after digestion (BF fraction), while very low levels of polyphenols were detected in the SW fraction.

As shown in Table 1, CW contains 0.84 mg/g of total polyphenols, largely gallic acid and hydroxycinnamic derivatives (HCD). As reported in [8] and because of unavoidable matrix interferences, simple phenolic compounds such as gallic acid could be not recovered in BF; however, a recovery higher than 45% was obtained for HCD in this fraction.

**Figure 1:**
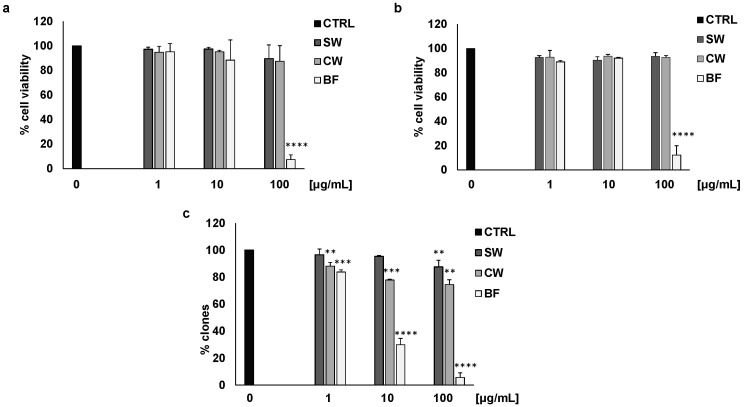
BF reduces colon cancer cell vitality and clonogenicity. HT29 (**a**) and HCT116 (**b**) cell vitality were evaluated by MTT assay after 48 h of cell exposure with FV extracts (1, 10, and 100 μg/mL). (**c**) Cell clonogenicity was reported as the percentage of colonies of HT29 cells in response to different concentrations (1, 10, and 100 μg/mL) of soaking water (SW), cooking water (CW), and the bioaccessible fraction (BF). **** *p* < 0.0001; *** *p* < 0.001 and ** *p* < 0.01 vs. basal.

Using two different colon cancer cell models (HT29 and HCT116), we evaluated cell viability by MTT assay. Soaking water (SW) and cooking water (CW) did not affect cell viability in basal conditions, while the bioaccessible fraction (BF) was strongly active at the higher concentrations (Figure 1a,b). These effects were not linked to a toxic effect of BF since trypan blue analysis indicated that 100 μg/mL of the extract did not induce significant cell death of the HT29 cell line (21.02 ± 6 cell death vs. 10.81 ± 4 of the blank). In addition, all the extracts inhibited HT29 colony formation, with BF being the most effective at all the tested concentrations (Figure 1c).

**Figure 2:**
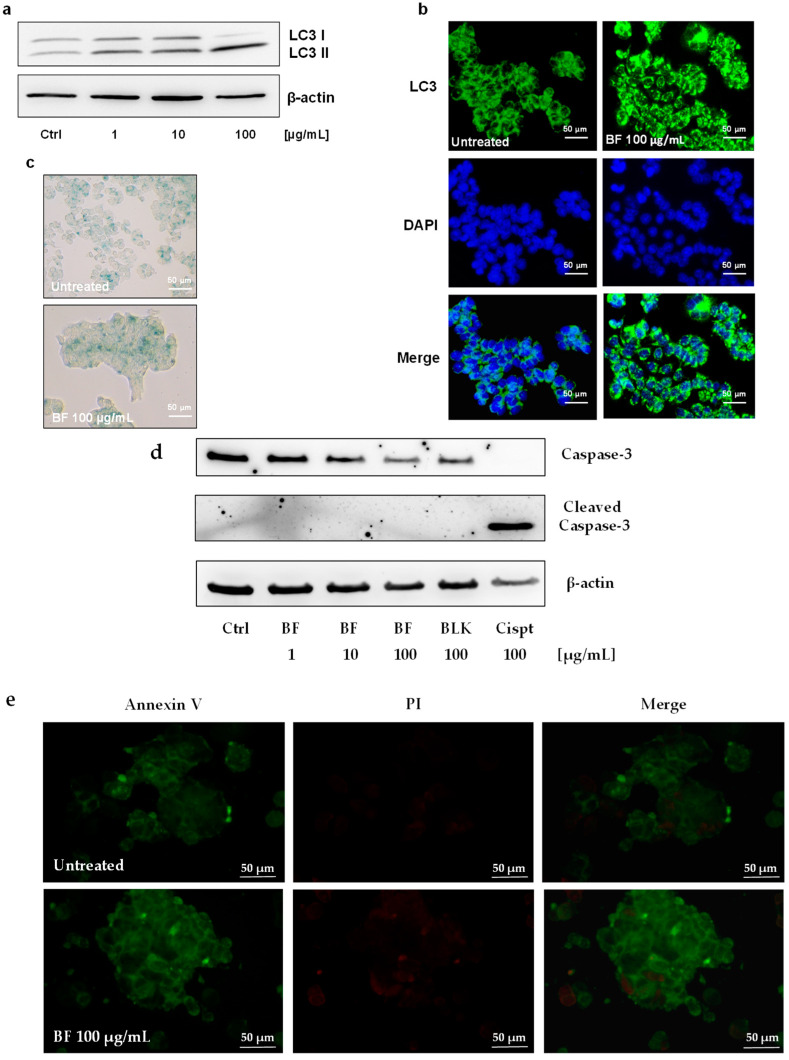
Autophagy activation by FV bioaccessible fraction (BF). HT29 cells were exposed to 1, 10, and 100 μg/mL of BF, and LC3 I and LC3 II expression and localization were evaluated by (**a**) Western blotting and (**b**) immunofluorescence analysis. (**c**) Senescence was evaluated in BF-treated HT29 cells. Senescent cells are colored in blue. HT29 cells were incubated with BF and apoptosis was evaluated by measuring caspase 3 activation through Western blotting (**d**) or by staining with annexin V-FITC-conjugated antibody and propidium iodide. Microscopy imaging was performed by using a 40× magnification (**e**).

To define the mechanism by which high concentrations of BF promoted the reduction in cell viability, we investigated its effects on the activation of autophagy, apoptosis, and senescence. Figure 2 shows that BF, at the concentration of 100 μg/mL, caused autophagy in HT29 cells by inducing LC3 II expression (Figure 2a,b). In addition, BF did not promote senescence (Figure 2c) nor activate the apoptotic process, since BF did not induce the activation of caspase 3 (Figure 2d) and annexin V (Figure 2e). These results confirm our previous observations concerning the biological activity of BF obtained from two other varieties of *P. vulgaris* [8].

### 3.2. Effects of P. vulgaris Extracts on Inflammation and Oxidative Stress Promoted by Interleukin 1β

It is broadly recognized that chronic inflammation, governing several pathologic conditions of the intestine including IBD [46], is the most critical contributor to the development and progression of cancer and that diet may modulate intestinal inflammatory processes [47,48]. In this scenario, we analyzed the activity of SW, CW, and BF on colon cancer cell vitality and growth promoted by interleukin 1β (IL1β), a potent proinflammatory cytokine. As HCT116 cells were very poorly responsive to IL1β treatment, we continued the experiments using only HT29 cells which proved to be more suitable for this study.

**Figure 3:**
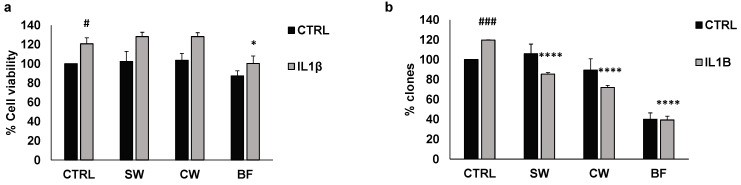
FV extracts inhibit HT29 cell vitality and clonogenicity induced by IL1β. (**a**) Cell vitality induced by IL1β was measured with or without FV extracts (10 μg/mL). (**b**) Cell clonogenicity was measured as the percentage of the number of HT29 colonies after treatment with IL1β and 10 μg/mL of soaking water (SW), cooking water (CW), andthe bioaccessible fraction (BF). # *p* < 0.05; ### *p* < 0.001 vs. basal and **** *p* < 0.0001; * *p* < 0.01 vs. IL1β.

HT29 treatment with 10 ng/mL IL1β caused increasing cell vitality that was reversed by BF (Figure 3a). In this type of experiment, all the bean extracts were used at the concentration of 10 μg/mL, the highest non-active concentration tested in basal conditions (Figure 1a). Similarly, IL1β resulted in an increase in the number of colonies, which was inhibited by SW, CW, and BF (Figure 3b). These data indicate that extracts from *P. vulgaris* var. Venanzio may be effective in reducing the protumoral effects of inflammatory mediators such as IL1β, and the digested fraction is the most active fraction.

**Figure 4:**
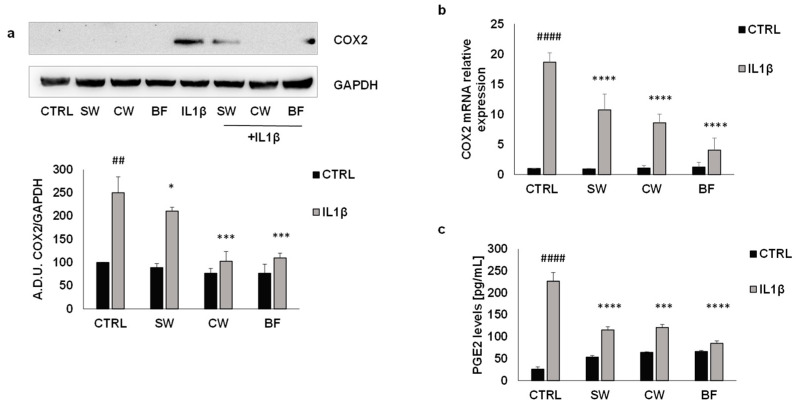
FV extracts inhibit COX2 expression and PGE2 production promoted by IL1β. COX2 expression was evaluated by (**a**) Western blotting and (**b**) PCR analysis in the HT29 cell line exposed to IL1β (10 ng/mL) with or without FV extracts (10 μg/mL). (**c**) Measurements of PGE2 levels by ELISA in HT29 cells treated with IL1β (10 ng/mL) in presence/absence of FV extracts (10 μg/mL). SW: soaking water, CW: cooking water, BF: bioaccessible fraction. PGE2 levels are reported as pg/mL. ## *p* < 0.01; #### *p* < 0.0001 vs. basal and **** *p*< 0.0001; *** *p* < 0.001; * *p* < 0.05 vs. IL1β.

To better understand the molecular mechanisms related to the inflammatory response of cancer cells, we evaluated the expression levels of cyclooxygenase 2 (COX2), an enzyme strongly involved in inflammation and tumor progression [49,50]. As expected, in HT29 cells, IL1β induced a potent increase in COX2 expression, evaluated by Western blotting, PCR, and measurement of PGE2 levels, which was reversed by all the FV extracts tested (Figure 4a–c).

**Figure 5:**
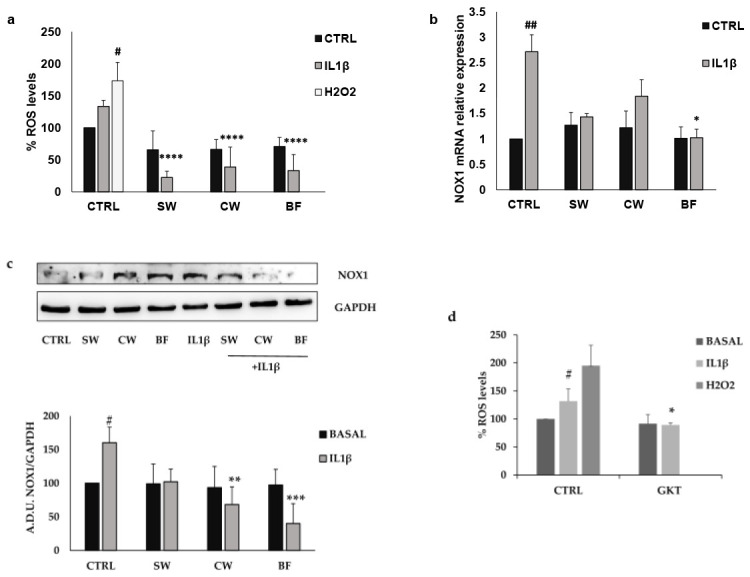
FV extracts inhibit ROS production and NOX1 expression induced by IL1β. (**a**,**d**) ROS measurement was performed after 24 h of incubation with IL1β (10 ng/mL) in the presence/absence of FV extracts (10 μg/mL) or GKT137831 (GKT) 10 μM. Data are expressed as % of ROS levels. (**b**,**c**) Expression levels of NOX1 were measured by PCR (**b**) and Western blotting (**c**). Cells were exposed to IL1β (10 ng/mL) in the presence/absence of FV extracts (10 μg/mL) for 24 h. Images are representative of three independent experiments. SW: soaking water, CW: cooking water, BF: bioaccessible fraction. ## *p* < 0.01; # *p* < 0.05 vs. basal and **** *p* < 0.0001; *** *p* < 0.001; ** *p* < 0.01; * *p* < 0.05 vs. IL1β.

Since inflammation is supported by oxidative stress, we evaluated the production of radical species of oxygen (ROS) after IL1β treatment. HT29 cells exposed for 24 h to 10 ng/mL IL1β showed an increased production of ROS, but when cancer cells were pretreated with SW, CW, and BF there was a significant reduction in ROS production that reached levels lower than baseline conditions (Figure 5a). Dysregulation of ROS production controls the development and progression of several types of cancer, including colorectal cancer, and the involvement of NADPH oxidases (NOXs) in ROS production appears to be of particular importance [51]. Among the seven members of the NOX family, NOX1 regulates the stemness, growth, and apoptosis of colon cancer cells via several mechanisms including the regulation of several oncogenes, chemokines, and angiogenic factors [52,53,54]. In this light, we measured the expression levels of NOX1 in the HT29 cell line both in basal conditions and in the presence of 10 ng/mL IL1β. Figure 5b,c show that basal expression of NOX1 in HT29 cells was increased after IL1β treatment. Consistently with the ROS measurements, BF reduced both NOX1 mRNA and protein expression levels, while the effects of SW and CW were limited to protein expression. The different effects of the fractions may be related to a different chemical composition.

**Figure 6:**
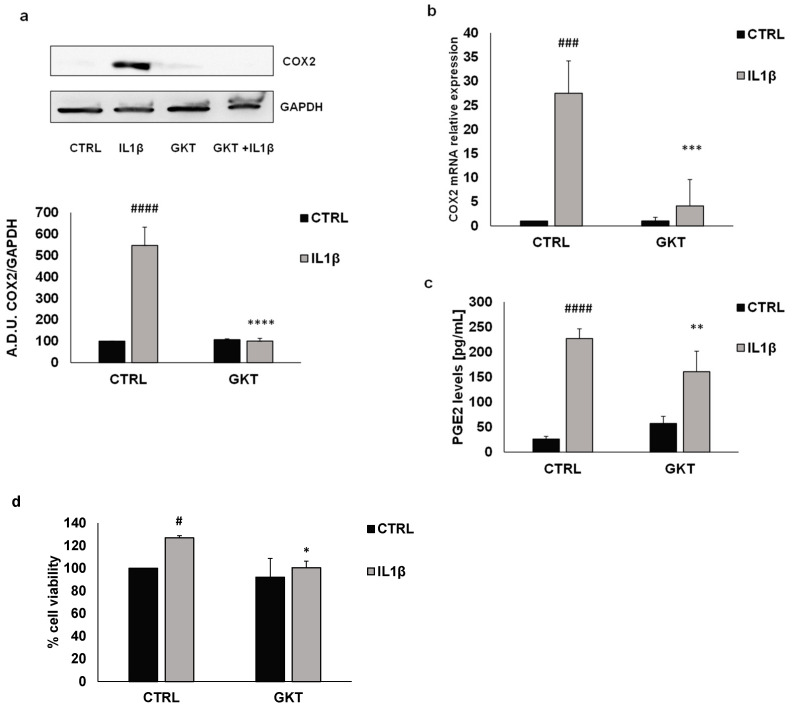
NOX1 activity regulates COX2 expression induced by IL1β in HT29 cells. COX2 expression induced by IL1β was evaluated by Western blotting (**a**) and PCR (**b**) after cell treatment with the NOX1 inhibitor GKT137831 (GKT) (5 μM). PGE2 levels (**c**), and cell viability (**d**) were measured in similar conditions. #### *p* < 0.0001; ### *p* < 0.001; # *p* < 0.05 vs. basal and **** *p* < 0.0001; *** *p* < 0.001; ** *p* < 0.01 and * *p* < 0.5 vs. IL1β.

As polyphenols are strongly reduced after the digestion phase (see Table 1), the activity of BF may be due to other chemical components, also of peptidic nature, produced during the digestive process. This will be the subject of further studies. These data suggest that FV extracts may modulate ROS production through NOX1 regulation. We observed a significant reduction in ROS production in IL1β-treated HT29 cells in the presence of the NOX1 inhibitor GKT137831 (GKT, 5 μM), confirming the importance of the NOX1 enzyme in redox balance dysregulation of colon cancer cells (Figure 5d).To better understand the correlation between COX2 induction and ROS production promoted by IL1β treatment, we performed a Western blot and PCR analysis of COX2 expression in the presence/absence of GKT (5 μM). As shown in Figure 6, GKT significantly prevented the protein and mRNA expression of COX2 and PGE2 production induced by IL1β, indicating that NOX1 expression and activity may be crucial for the expression of inflammatory markers in colon cancer cells. Consistently, cell viability promoted by IL1β was inhibited by GKT (Figure 6d).

### 3.3. P. vulgaris Extracts Inhibit IL1β Effects in Colon Cancer Cells Amplified by PGE2

The role of inflammation in the onset and progression of cancer is widely recognized, and an important role is covered by PGE2 [49,50]. This prostaglandin can be produced by cancer cells themselves (intrinsic inflammation) and by the tumor microenvironment (extrinsic inflammation), and it may promote growth, migration, angiogenesis, and immunoescape [49,50].

**Figure 7:**
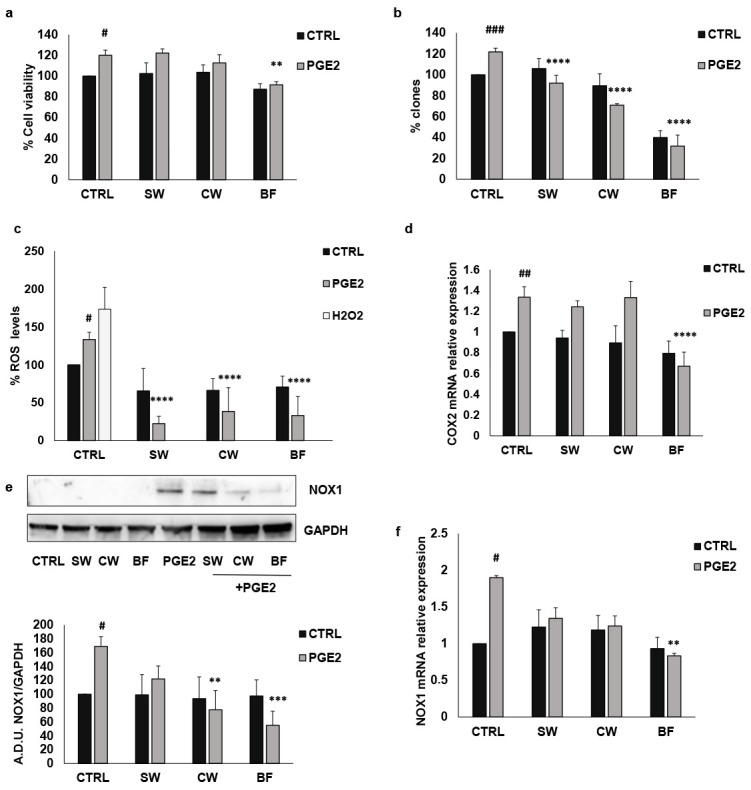
FV extracts inhibit PGE2 activity. (**a**) Cell vitality induced by PGE2 (1 μM) was measured in the presence/absence of SW, CW, and BF (10 μg/mL) by MTT assay. (**b**) Cell clonogenicity was reported as the percentage of colonies of HT29 cells in response to PGE2 and SW, CW, and BF (10 μM). (**c**) ROS measurement was performed after 24 h of incubation with PGE2 in the presence/absence of FV extracts. (**d**) COX2 mRNA levels were measured after 24 h of exposure to PGE2 and FV extracts. (**e**,**f**) Expression levels of NOX1 were measured by Western blotting and PCR. Cells were exposed to PGE2 in the presence/absence of FV extracts (10 μg/mL) for 24 h. SW: soaking water, CW: cooking water, BF: bioaccessible fraction. ### *p* < 0.001; ## *p* < 0.01; # *p* < 0.05 vs. basal and **** *p* < 0.0001; *** *p* < 0.001; ** *p* < 0.01 vs. IL1β.

The treatment of HT29 cells with IL1β for 24 h led to increased COX2 expression and PGE2 production (Figure 4). To verify the influence of *P. vulgaris* extracts on PGE2 effects, we first evaluated the effects of SW, CW, and BF on cell growth prompted by PGE2. As observed for IL1β, PGE2 promoted both HT29 cell viability, which was significantly inhibited by the highest BF concentration, as well as colony formation, which was reduced by all the extracts used (Figure 7a,b). Similarly to IL1β, PGE2 also promoted ROS production and NOX1 and COX2 expression, which were inhibited when colon cancer cells were pre-treated with SW, CW, and BF (Figure 7c–f), indicating that the bean extracts also inhibited the effects of exogenous PGE2. These data support the hypothesis that *P. vulgaris* extracts modulate the effects of inflammatory mediators through the reduction in ROS production and inflammatory pathways.

## 4. Discussion

Inflammation is characterized by a series of biological responses of the body to dangerous events and represents a physiological process that leads to the resolution of different critical events (acute inflammation) [55]. The process transforms from physiological to pathological when it is not conclusive and persists over time. In this case, chronic inflammation is associated with damaging diseases, namely chronic inflammatory diseases [55]. Among these types of pathological conditions, IBD is a chronic inflammatory disease of the digestive tract [48] and may lead to the development of colon cancer [27,28]. In fact, starting from Virchow, it is now widely recognized that chronic inflammation is closely related to the onset and progression of many tumors through the promotion of growth, migration, invasion, apoptotic escape, angiogenesis, and metabolic reprogramming of cancer cells [56,57,58]. Thus, inflammatory stimuli promote cancer cell growth by activating intracellular signaling pathways.

In this context, the reduction in chronic inflammation through diet may represent an important endpoint, and correct eating behavior, including more vegetables and less saturated fats, supports the prevention and management of pathological disorders. Moreover, there is growing evidence that specific nutritional patterns may interfere with the progression of chronic diseases. These observations are in line with an increasing need for dietary approaches and functional foods that may be useful to treat these conditions. In line with these observations, we report experimental evidence regarding the effect of *P. vulgaris* extracts on the inflammatory pathways involved in intestinal inflammation and colon cancer progression. Bean extracts inhibited colon cancer cell growth promoted by IL1β and PGE2, both involved in chronic inflammation. Consistently, the extracts inhibited COX2 expression. Interestingly, we showed that there is a relationship between ROS production and COX2 expression, NOX1 being responsible for the induction of COX2 promoted by IL1β and PGE2. These data indicate that food may affect cellular signaling pathways that contribute to disease onset and progression, and further highlight the importance of diet in human health.

Importantly, we evaluated the biological activity of a new variety of *P. vulgaris*, the Fagiola di Venanzio (FV). This variety was only recently recognized as a new local endangered bean variety and represents a local source of biodiversity [2]. In a world where the decline of biodiversity is increasingly rapid, biodiversity preservation should be a global goal and a new strategy for the promotion of health for both people and nature is required. Local foods represent a new source of bioactive compounds and are assuming a prominent role in ensuring public health. For this reason, our study on the Fagiola di Venanzio represents a particularly contemporary topic.

A critical point in characterizing the food’s biological activity is the procedure used to prepare the extracts. The lack of physiological conditions during extract preparation that do not consider the digestive processes within the human gastrointestinal tract may represent a limiting factor in understanding the biological role of some foods. To produce a bean extract that simulates the one produced by the digestion process (bioaccessible fraction, BF), we used an in vitro procedure that, taking into account digestive enzymes, pH, salt concentration, and digestion time typical of the different phases of digestion in the gastrointestinal tract, allowed us to simulate physiological conditions [8].

Another important aspect is that we examined the activities of three different extracts, each representing a crucial step in the processing of the beans: soaking water, cooking water, and the bioaccessible fraction obtained after in vitro enzymatic digestion. Soaking water was obtained after keeping the beans in water overnight. This extract, representing the first step of bean processing, is not normally consumed but constitutes a significant waste product of the large-scale industrial production of cooked beans that needs to be disposed. We demonstrated that soaking water may be a good source of bioactive compounds and represents a huge opportunity for the bioconversion of bean waste into useful materials to be considered in the context of the circular economy. In this paper, we have not reported the chemical composition of this fraction as the analysis is still ongoing. However, SW was very scarce in polyphenols, and we hypothesize that the main constituents of this waste product may be lectins, as reported for other varieties of beans [59]. The major limitation of this study is represented by the characterization of the chemical composition of the different bean extracts, which, at the moment, is limited to the determination of the polyphenol content in all the fractions. However, we expect the bioaccessible fraction of beans to contain degraded complex sugars and oligopeptides [60,61,62]. Thus, BF requires comprehensive chemical characterization which is beyond the scope of this paper. Indeed, it is made difficult by the inevitable analytical interferences after simulated digestion and the technical challenge required to determine the chemical profile of a digested phytocomplex.

## 5. Conclusions

We studied the biological activity of different extracts of a specific variety of *P. vulgaris*, including the fractions obtained after mimicking the traditional cooking procedure and gastrointestinal digestion. The different fractions reduced cancer cell clonogenicity and inhibited COX2 expression and activity induced by IL1β. Consistently, the tested extracts reduced oxidative stress by counteracting IL1β’s effects on the expression of NOX1 and the production of ROS. These antioxidant and anti-inflammatory activities support the idea that FV consumption may protect against inflammatory diseases such as IBD and CRC.

These data support the importance of foods in human health and the pivotal role played by local food biodiversity.

Furthermore, from a circular economy perspective, we propose a possible use of a waste product as a source of bioactive compounds to be used for the preparation of food supplements as well as in pharmaceutical and nutraceutical preparations.

## Figures and Tables

**Table 1:** Polyphenol content of CW and BF extracts of FV beans. Total polyphenols were quantified by colorimetric analysis and expressed in mg/g dry weight of raw beans, as gallic acid equivalents. Hydroxycinnamic derivatives were identified according to their UV spectra by HPLC-DAD and quantified as chlorogenic acid (mg/g dry weight). n.d.= not detectable.

Components	CW	BF
Total polyphenols	0.84 ± 0.01	n.d.
Total hydroxycinnamic derivatives	0.24 ± 0.01	0.11 ± 0.01
Gallic acid	0.41 ± 0.02	n.d.
Chlorogenic acid	0.03 ± 0.01	n.d.

## Data Availability

The original contributions presented in the study are included in the article, further inquiries can be directed to the corresponding author.
